# Characterization of a Sulfonated Polycarbonate Resistive Humidity Sensor

**DOI:** 10.3390/s130202023

**Published:** 2013-02-05

**Authors:** Carla P.L. Rubinger, Hallen D.R. Calado, Rero M. Rubinger, Henrique Oliveira, Claudio L. Donnici

**Affiliations:** 1 Departamento de Física e Química, Instituto de Ciências, Universidade Federal de Itajubá, CP 50, 37500-903, Itajubá-MG, Brazil; E-Mail: rero@unifei.edu.br (R.M.R.); 2 Departamento de Química, ICEx, Universidade Federal de Minas Gerais, 31270-901, Belo Horizonte-MG, Brazil; E-Mails: hallendaniel@yahoo.com.br (H.D.R.C.); henrique.bh@hotmail.com (H.O.); cdonnici@terra.com.br (C.L.D.)

**Keywords:** sulfonation, humidity sensors, impedance spectroscopy

## Abstract

In this work; resistive moisture sensors were obtained by dip coating sulfonated polycarbonate (SPC) onto silver interdigitated electrodes. Commercial polycarbonate was sulfonated with acetyl sulphate at two different sulfonation degrees corresponding to 9.0 and 18.0 mole %. Impedance spectroscopy was used to investigate the humidity sensing properties at controlled relative humidity (RH%) environments generated from standard saline solutions in the range of 11–90 RH%. For the highest sulfonated sample; in the RH% range investigated (11 to 90%); the sensor impedance changed from 4.7 MΩ to 18 kΩ. Humidity sensors made from sulfonated polycarbonate showed exponential decay behavior of the impedance at constant frequency with the environmental relative humidity. Sample 9SPC presented dielectric relaxation response for environmental humidity between 58 and 90 RH% while sample 18SPC presented dielectric relaxation response for the entire measured range between 11 and 90 RH%. Sulfonated polycarbonate could be a promising material for the fabrication of simple and cheap humidity-sensing sensors for the assessment of relative humidity of the surrounding environment, as suggested by experimental results.

## 1. Introduction

Humidity detection and monitoring is important in fields such as weather, agriculture, industrial, household electric appliances, medical field and research [1–3]. The production of a good humidity sensor is complex since high-performance humidity sensors must meet several requirements, including linear response, high sensitivity, fast response time, chemical and physical stability, broad operating range of humidity, and low cost [4]. Polymers, ceramics and composites with such characteristics have been extensively studied [5].

There are many kinds of humidity sensors. Capacitive humidity sensors involve a film deposited between electrodes on a substrate. The dielectric constant is proportional to the relative humidity and changes due to water steams [6–9]. The properties of capacitive sensors depend on the electrode area and the gap between the electrodes [6,7]. Resistive humidity sensors measure the change in the electrical resistance of a sensor due to proton conduction and the sensor resistance is inversely proportional to relative humidity. Protons are obtained by electrolysis of the water bound to the ionic hygroscopic groups which increases with relative humidity. Most resistive humidity sensors have LiCl as the sensing element [6], and the operating principle is based on ionic conductivity [7]. This device is very sensitive to temperature; several efforts have been made to improve this device by using the conductance technique [6]. Some organic materials exhibit good response to moisture, but dissolve in water [10–12] and also present undesired limitation in the detection of environment for humidity lower than 20% RH [13]. Organic materials that are insoluble in water such as cellulose acetate butyrate and polyimide have been used as humidity sensors [12]. Some recent works on polymeric humidity sensors have added an electronic conducting channel that does not respond to relative humidity. For instance in [13] polypyrrole and in [14] carbon nanotubes are used. For most of the relative humidity range, these materials do not contribute to enhance sensing properties since they just add an electronic conducting channel in parallel, *i.e.*, a resistor that is parallel to the real proton conducting channel. Such a resistor will reduce the impedance range change which means lower sensitivity. By inserting a conductive material on the humidity sensor matrix this will allow protons with very short diffusion lengths to be collected before being bound to an ionic group inserted in the polymeric matrix. Such sensors will behave with improved response for low humidity but at a price to drastically reduce the response at mean and high humidity.

In the present work, we opted to use a simple proton conducting membrane with improved humidity range sensitivity compared to similar materials. Besides the fact that this guaranties a high sensitivity (*i.e.*, exponential change of impedance by many magnitude orders) on the academic point of view the transport of protons on a membrane is not really a simple process and deserves to be investigated without the influence of other processes. Sulfonated polycarbonate with sulfonic acid groups to the degrees of 9.0 and 18 mole % are also insoluble in water and this turns it into interesting to be investigated as humidity sensor. In a previous work, sulfonated polystyrene (SPS) was investigated as a humidity sensor [15]. For this material, the sensor impedance presented exponential dependence with the environmental relative humidity in a however narrow range than that of the present work [15].

Due to better mechanical and chemical properties, *i.e*., its resistance to impact, sulfonated polycarbonate (SPC) has already been evaluated for some applications such as membranes for fuel cell [16], for separation of mixtures of carbon dioxide and methane [17], and for separation of inorganic solutes and dyes from aqueous solutions [18]. Composites of polypyrrole-SPC [19], polyaniline-SPC [20], SPC-polyvinylidene fluoride [21] were also investigated. In such cases, the sulfonic group was introduced into the structure of the polycarbonate (PC) in order to enhance the coulomb interaction between each phase of the composite [19,20].

In the present work, sulfonated polycarbonate, SPC, with the sulfonation group SO_3_H was investigated as a relative humidity sensor. The preparation of humidity sensors was considered for SPC at two different sulfonation degrees: sensors 9SPC and 18SPC corresponding to 9.0 and 18.0 mole %. The investigations of their humidity sensitivity, vibrational and morphological characteristics are here reported. In order to obtain the sensor impedance dependence with RH% at the frequency range dominated by the resistive behavior, we selected from impedance spectroscopy measurements (carried out from 5 Hz to 13 MHz) the ac frequencies of 25 Hz and 200 Hz for sensor 9SPC and 18SPC respectively. Considering relative humidity generated from standard saline solutions in the range of 11–90 RH%, our previous work in which a 22 mole % SPS sensor presented resistive behavior in the range of 33–90 RH% [15] and, in the present work, sensor 9SPC investigated at 25 Hz, presented resistive behavior within the range of 58% to 90 RH% whilst sensor 18SPC investigated at 200 Hz have shown resistive response in a wider relative humidity range, *i.e.*, in the range of 11–90 RH%.

## 2. Experimental Section

### 2.1. Materials and Methods

The starting polymer was a polycarbonate (PC) (from Policarbonatos do Brasil S.A., São Paulo, Brazil) with $\overset{̅}{M_{n}}$ = 8.1 × 10^4^g.mole^−1^ and $\overset{̅}{M_{w}}$/ $\overset{̅}{M_{n}}$ = 3.8, used as received. Sulfuric acid (95–97%, Merck,), acetic anhydride (Synth, P.A.), dichloromethane (Synth, P.A.), 2 propanol (Synth, P.A.; were used as received without further purification.

SPC was obtained by sulfonation of commercial PC in CH_2_Cl_2_ at 40 °C with acetyl sulfate, following the method described for the polystyrene [15]. The sulfonation level of SPC was determined by titration following the method described by Smitha *et al.* [16]. The sulfonation degree (x) is expressed as mole percent, xSPC. In this work, the sulfonation levels achieved and used were 9.0 and 18.0 mole % of the sulfonated carbonate repeating units.

**Figure 1.**
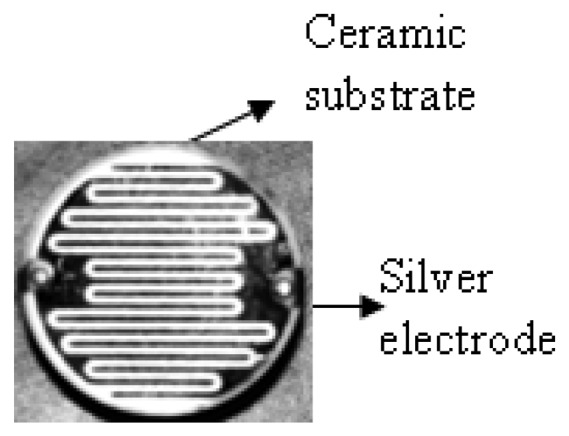
Architecture of the humidity sensor device. The SPC film is deposited over the electrodes.

The preparation of solutions for PC/CH_2_Cl_2_ (0.05 g/mL), 9SPC/CH_3_OH and 18SPC/CH_3_OH (0.05 g/mL) films were followed by stirring to give homogenous solutions. Each solution was deposited by dip coating on insulating disk-shaped ceramic substrate of 25.0 mm diameter, inert with respect to humidity changes, where an interdigital array of silver electrodes has been defined (Figure 1). The thickness of both films was (400 ± 50) nm. Films are stable at least for six months.

A set of RH% environments were obtained with saturated aqueous solution of salts: LiCl 11%, MgCl_2_ 33%, K_2_CO_3_ 44%, NaBr 58%, NaCl 75%, KCl 84% and BaCl_2_ 90%. For each humidity value, the sensors were placed in the recipient for half an hour to enable the humidity source to reach equilibrium before measurements began.

### 2.2. Characterization

Infrared spectra were recorded using a Perkin-Elmer FTIR 1600 spectrophotometer. Spectra were obtained in the mid-infrared region (500–4,000 cm^−1^) and for samples prepared in KBr pellet (2 mg of polymer into 300 mg of KBr) following standard procedures. The FTIR spectra were used to qualitatively characterize the ∼SO_3_H groups. The surface morphology of the SPC deposited by dip coating was studied by scanning electron microscope (SEM) in JEOL Model JSM T300 equipment, operating at 20 kV. The samples were gold-sputtered prior to the measurements. The impedance measurements were done with an HP4192A impedance analyzer. The experimental parameters were: frequency range of 5 Hz to 13 MHz, sinusoidal voltage amplitude = 1.0 Vrms. All the measurements were carried out at a room temperature (*i.e.*, 30 °C).

## 3. Results and Discussion

**Figure 2.**
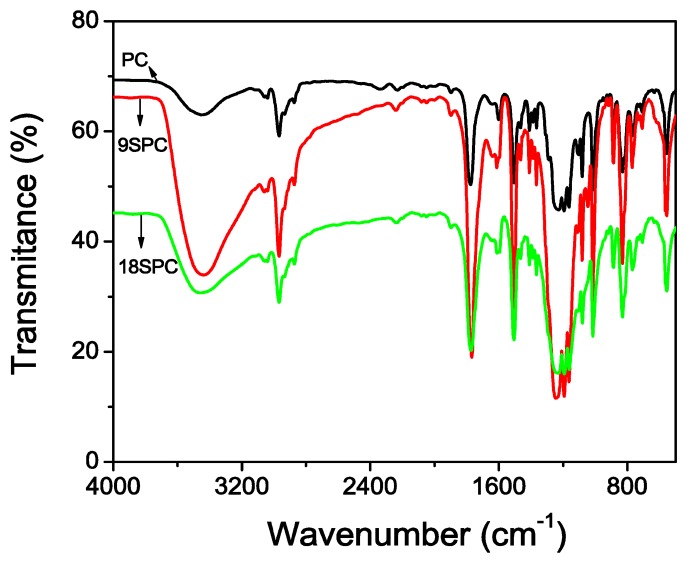
Infrared spectra of PC, 9SPC and 18SPC.

Qualitatively, polycarbonate sulfonation was verified by infrared spectroscopy. Figure 2 shows the results of FTIR spectra for both SPC materials from 250 to 4,000 cm^−1^. The detection of a broad band at approximately 3,408 cm^−1^ has been ascribed to stretching modes of hydroxyl groups of SO_3_H groups. The vibrational modes of SO_3_H are observed at 1,257 cm^−1^ and 1,030 cm^−1^. The vibrational modes at 1,257 cm^−1^ can be ascribed to stretch vibration for S = O and that one at 1,030 cm^−1^ is assigned to the symmetric stretching band. However, the stretching band of carbonyl group is 1,766 cm^−1^ in the ester group for SPC. Thus, FTIR data indicates the presence of SO_3_H groups, which confirms the occurrence of sulfonation [21].

**Figure 3.**
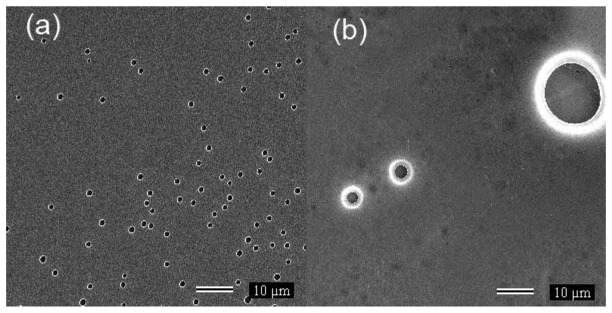
(**a**) SEM micrograph of film PC. (**b**) SEM micrograph of film 18SPC.

Figure 3(a,b) present SEM images of PC and 18SPC samples, respectively. The gray regions indicate polymer films whereas the black regions indicate the mica substrate. The PC film (Figure 3(a)) is flat with some circular pores, probably due to the presence of micro bubbles during solvent evaporation. Although not shown, there are large areas without bubbles implying a good regularity of the PC film. 18SPC film image (Figure 3(b)) is mostly flat but with large bubbles. The porosity and surface irregularities of the films may increase the contact area to the ambient moisture which could increase the water absorption rate [22,23]. Therefore, the water absorption by SPC films depends to the sulfonation degree due to the water affinity of the SO_3_H group, as will be demonstrated by impedance measurements.

**Figure 4.**
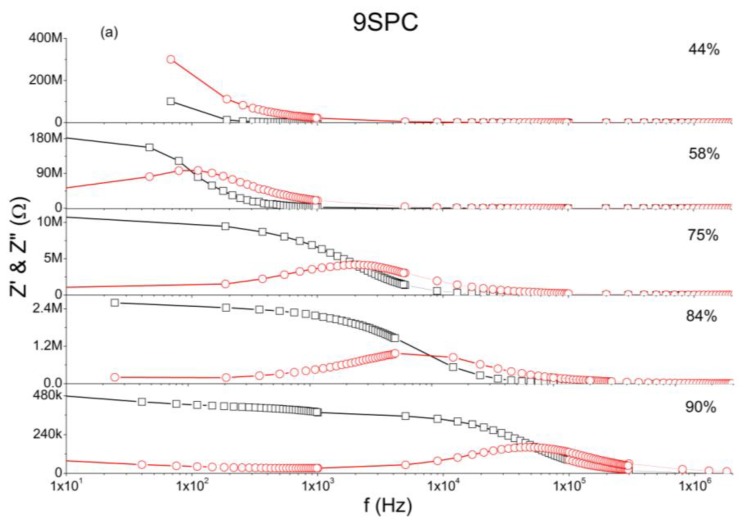

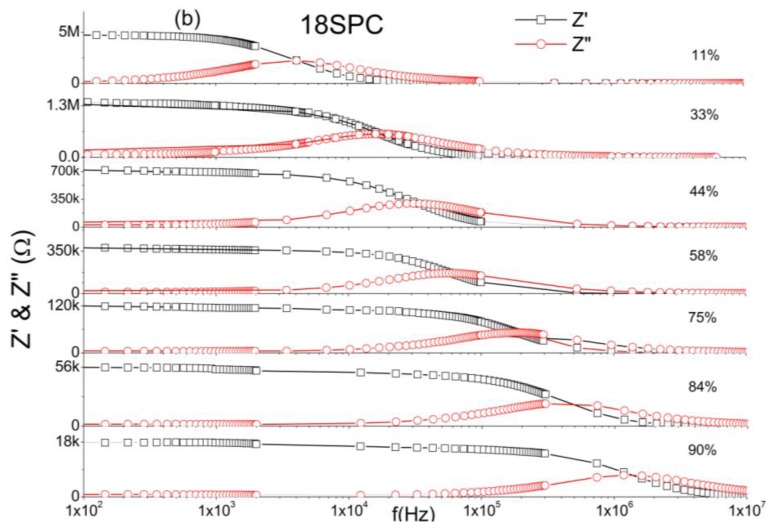
Real (Z′ square symbols) and imaginary (Z″ circles) parts of the impedance *versus* frequency, for various relative humidity values. All measurements were presented in the same horizontal scale as function to the log of the frequency. The straight horizontal lines were employed to separate the different RH% environments. The results were presented in: (**a**) for the sample 9SPC and (**b**) for the sample 18SPC.

**Figure 5.**
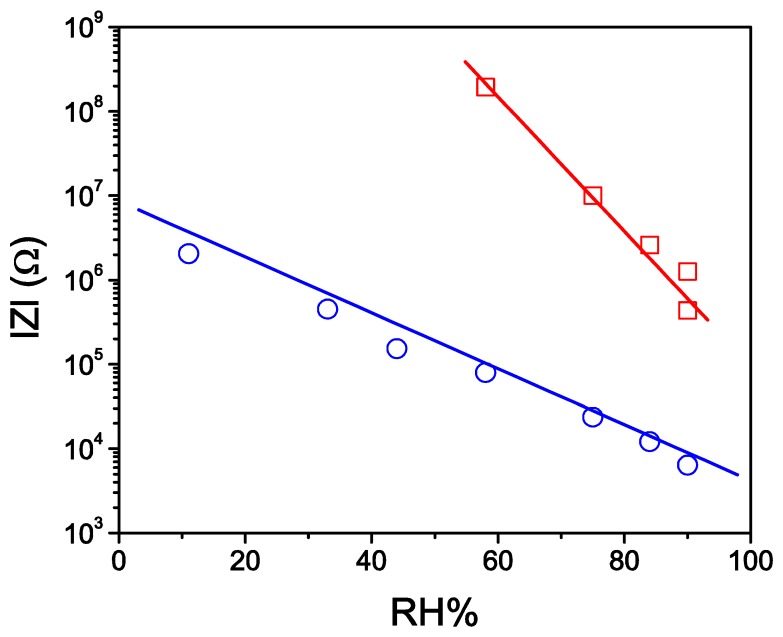
Dependence of the logarithm of impedance modulus from the relative humidity of films 9SPC (red squares) and 18SPC (blue circles) at 25 Hz and 200 Hz, respectively. The solid lines correspond to the fittings.

The analysis of complex impedance is important tool to investigate the sensing behavior of humidity sensors. In Figure 4 at low frequency region of impedance measurements is observed that, the impedance is almost constant within a wide frequency range. In this case, a measurement at a single frequency would be meaningful to indicate moisture level as shown in Figure 5.

Figure 4(a) shows that the main result is that at low frequencies the impedance of 9SPC changes from around 195 MΩ to about 465 kΩ for 58% to 90% of relative humidity respectively (RH%). Figure 4(b) shows that the main result is that at low frequencies the impedance of 18SPC changes from around 4.7 MΩ to about 18 kΩ for 11% to 90% RH% respectively. In this humidity range and at low frequencies the impedance is mostly resistive and practically constant over a wide frequency range. For sample 9SPC, at 44% of RH% impedance is even higher, but the capacitive (imaginary) part predominates meaning that it does not present a broad frequency range with constant impedance.

At the whole relative humidity range the measurements present relaxation phenomena for sample 18SPC and, for sample 9SPC, only within the range of 58% to 90 RH%. For sample 9SPC, at 44% of RH% carriers do not reach the opposite electrode before bounding on an ionic group, leading to very high impedance, similar to the PC material [24].

Regarding the change of the impedance magnitude order in the relative humidity range considered in Figure 5, it decreases by a factor of three orders for sample 9SPC and of two orders for sample 18SPC.

Measurements of impedance modulus from the relative humidity of films 9SPC and 18SPC at 25 Hz and 200 Hz, respectively, were carried out at temperatures of 20 °C, 30 °C, 40 °C and 50 °C. It was observed that the impedance modulus is practically constant within this temperature range. For this reason graphical results for temperature dependence will not be shown.

Furthermore, two processes are responsible for impedance decreasing: the first is associated with the water electrolysis and the second is related to the transport of H^+^ ions [25]. This transport has diffusive nature at low frequencies. The presence of absorbed water in the polymer allows not only the electrolysis, but also reduces the ionic interaction between the polar groups of the polymer and the H^+^ ions, which in turns increase the ion mobility [20]. Therefore, with mobility increasing the impedance decreases, and it is observed experimentally. According to the references [24–27] the absorption of water at constant temperature occurs due to the interaction between water molecules and charged sites (here represented by SO_3_H groups).

For the polyelectrolyte humidity sensors, the conduction mechanism is mainly ionic, and sensing behavior depends on both the kind and concentration of ions and the water absorption ability of the sensing film [28].

The electrical transport phenomenon in polymeric humidity sensors is yet to be fully described. There is much work to carry on in understanding the fundamental physical phenomena presented in such sensors. For instance, protons have high mass and very low mobility and d.c. measurements are impossible to be carried out due to double layer capacitance effects. Under this scenario, Hall effect measurements cannot be easily carried out and due to limitations of water melting and boiling points, temperature dependence of resistivity can be measured in a quite short range, which turns the determination of activation energies associated with carrier statistics a hard task.

Despite the limitations, an empirical model of impedance exponential decay with relative humidity can lead to reasonable results and was applied to the sensor impedance as a function of RH%. Figure 5 presents the impedance modulus, in a log scale and measured at 25 Hz for sample 9SPC and 200 Hz for sample 18SPC as a function of relative humidity. The observed behavior in a log scale is linear, which could lead to an empirical functional dependence of the type:

(1) $$Z=Z_{0}\cdot 10^{-B•RH\%}$$

where Z_0_ is the projected impedance at 0 RH% and B is the slope obtained from fitting. From the fittings of data for sample 9SPC were obtained Z_0_ = (10 ± 5) TΩ and B = (0.080 ± 0.006) RH%^−1^ and for sample 18SPC Z_0_ = (13 ± 1) MΩ and B = (0.029 ± 0.002) RH%^−1^.

**Figure 6.**
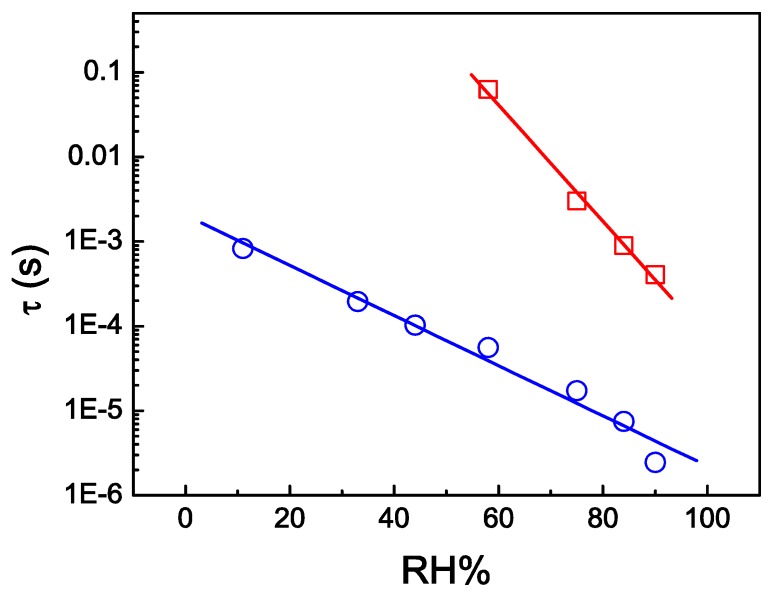
Dependence of the logarithm of relaxation time from relative humidity of films 9SPC (red squares) at 25 Hz and 18SPC (blue circles) at 200 Hz.

Another form of measuring RH% would be through relaxation times obtained from impedance spectroscopy measurements, as represented in Figure 6. The main characteristic response for the SPC sensor is that the resistive regime broadens with increasing humidity. As a consequence the relaxation shifts to higher frequencies. The characteristic relaxation time τ, obtained from the condition 2πf_MAX._τ =1 (*i.e.*, at the maximum of Z″), reduces with increasing RH% (Figure 6). This relaxation time is associated with dipole polarization. Sample 9SPC and 18SPC have distinct peak frequencies of relaxation. For sample 9SPC the range is between 10^2^ and 10^5^ Hz for 58 to 90 RH% respectively and or sample 18SPC the range is between 10^3^ and 10^6^ Hz for 11 to 90 RH% respectively.

Comparing Figures 5 and 6 one can identify a good similarity for the RH% functional dependence. This form of measuring the relaxation regime considers expensive equipment, while measuring at a fixed frequency of 25 Hz or 200 Hz would be inexpensive to implement with a few integrated circuits and discrete electronic components. Thus, the similarity between Figures 5 and 6 proves the efficiency of the fixed frequency method proposed. Finally, it is interesting to report that SPC RH% electrical characteristic dependence turns it into a promising resistive humidity sensor.

The 18SPC sensor presented relaxation for humidity as low as 11 RH%. Measurements salt solutions for RH% lower than 11% have not been prepared, but the sensing properties could work even for humidity lower than 11 RH%.

Regarding sample 9SPC the measurements for humidity lower than 58 RH% presented a hyperbolic behavior of the Z″ with frequency, similar to a pure dielectric behavior. One possible explanation for the observed results is the reduction of the diffusion length to a distance smaller than the contact distance. Thus the absorbed water and H^+^ ions would bind to SO_3_H groups before reaching the opposite contact, increasing enormously the sensor impedance. This was previously proposed for a humidity sensor [17].

The absorption (changing the environment from 11 RH% to 90 RH%) and desorption (changing the environment from 90 RH% to 11 RH%) times for both SPC sensors were carried out. One consideration is that about 30 s was used to change from one environment to the other and to start the measuring program. For sample 9SPC both absorption and desorption times are low (at least lower than the 30 s lost during the environment change). For sample 18SPC a low absorption time and desorption time of 8 min after the changing time was measured.

## 4. Conclusions

Resistive moisture sensors were obtained by dip coating sulfonated polycarbonate onto silver interdigitated electrodes. The SPC was sulfonated to a degree of 9 mole % (9SPC) and 18 mole % (18SPC). Humidity sensors made from SPC show exponential dependence of the impedance with the environmental relative humidity. An empirical model of exponential decay of the impedance measured at fixed low frequencies with the relative humidity was used to fit experimental results. This model allows practical conversion between impedance and relative humidity. Sample 9SPC presented dielectric relaxation response for environmental humidity between 58 and 90 RH% while sample 18SPC presented dielectric relaxation response for the entire measured range between 11 and 90 RH%. The uncertainties of the relative-humidity are 8% and 4% for samples 9SPC and 18SPC respectively. The sensor of 9SPC is suitable for applications that consider high humidity environments and the need of quick responses. The sensor of 18SPC is suitable for applications that consider broad range of humidity environments. These results turn the sulfonated polycarbonate into a very promising material for the fabrication of inexpensive and efficient humidity sensors.
